# Clinical, Biochemical, and Radiological Correlation in the Severity of Acute Pancreatitis: A Retrospective Study

**DOI:** 10.7759/cureus.34996

**Published:** 2023-02-14

**Authors:** Rahul Saxena, Shishir Kumar, Zaid Nafe, Ashok Chattoraj, Shivraj Chauhan

**Affiliations:** 1 Department of General Surgery, Tata Main Hospital, Jamshedpur, IND; 2 Department of Gastroenterology, Tata Main Hospital, Jamshedpur, IND; 3 Community Medicine, Dr. Vaishampayan Memorial Government Medical College (VMGMC), Jamshedpur, IND

**Keywords:** alcohol, necrotizing pancreatitis, epigastric tenderness, pancreas, acute pancreatitis

## Abstract

Objectives: To analyze the etiologies and the varying clinical presentations and to validate the clinical, biochemical, and radiological signs with severity and prognosis of acute pancreatitis.

Methods: A retrospective study of 1316 patients diagnosed with acute pancreatitis in an industrial hospital in Jamshedpur, Jharkhand, was conducted, and their clinicoradiological profiles, etiological factors, and outcomes were studied.

Result: A total of 1316 cases were enrolled, out of which maximum cases (411 [31.23%]) were from the age group of 30-44 years, and the mean and median age were 44.54 and 47 years, respectively. A total of 731 (55.45%) patients had social habits (i.e., alcohol and smoking), and 585 (44.45%) patients did not have any social habits. Based on the etiology of acute pancreatitis (AP), the majority of cases were due to alcoholism (710 [53.95%]) followed by gallstone (343 [26.06%]) and idiopathic pancreatitis (217 [16.48%]). As per the severity of AP, most patients showed mild pancreatitis (937 [71.20%]) followed by moderate (312 [23.71%]) and severe pancreatitis (67 [05.09%]). Mild and moderate pancreatitis patients were shown in 85 and 28 cases, respectively, suggestive of chronic pancreatitis after repeated episodes of AP. But severe pancreatitis shown in 19 cases had hypocalcemia + shock + multi-organ dysfunction syndrome (MODS). In mild, moderate, and severe AP, the mortality rates were 19 (02.03%), 44 (14.10%), and 21 (31.34%), respectively. Overall, 1232 (93.62%) of AP cases recovered and were discharged in stable condition, but 84 (06.38%) cases expired.

Conclusion: AP is a common cause of acute abdomen in patients presenting to the surgical emergency department. The management is mainly conservative with surgery limited to only a few selected cases, depending upon the severity of the disease.

## Introduction

In his literature, Eristratos (310-250 BC) used the phrase "pancreatic." In 100 AD, Rufus of Ephesus coined the term "pancreas." Pan (a Greek word, which means "all") refers to the entire organ because it lacks both cartilage and bone [1]. Acute pancreatitis (AP) is characterized as a sudden onset of pancreatic inflammation with varied involvement of nearby tissues or distant organ systems. The histology may return to normal between bouts, or it may manifest as a solitary attack or reoccur in discrete episodes [2]. It has a wide range of signs and symptoms, from those associated with mild, self-limiting illnesses to those associated with fulminant conditions that cause multi-organ failure and significant mortality [2]. AP has a mortality rate of 2%-10% overall. In particular, this relates to 10%-30% of patients with severe illness that is characterized by pancreatic and peripancreatic necrosis. Together, alcoholism and gallstones cause 80% of AP. Most of these individuals had mild to moderately severe pancreatitis that was treatable with conservative measures, and they fully recovered. Only 15% of individuals experienced severe AP [3]. AP can be identified using computed tomography (CT) scans and ultrasounds. Ultrasonography is not a sensitive diagnostic because adipose tissue and intestinal gas may cover the pancreas in over one-third of patients. However, gallstones, bile duct stones, and bile duct dilatation can all be found with ultrasound extremely well [4]. The most accurate test for identifying AP is CT. Pancreatic necrosis can be accurately diagnosed with contrast-enhanced CT. Pancreatic necrosis can be accurately predicted by dynamic CT, which is accomplished by rapidly injecting massive volumes of intravenous contrast [5].

Interstitial edema is the main radiologic feature of mild AP. White blood cells, primarily neutrophils, are abundantly visible under a microscope in the interstitial space. Small foci of acinar cell necrosis occasionally can be identified, despite the fact that parenchymal necrosis is not overtly evident. Adipose tissue within and around the pancreas frequently demonstrates necrosis. The macroscopical signs of severe AP include substantial alterations in the peripancreatic fat necrosis and turbid, hemorrhagic fluid in the peritoneal cavity. At the microscopic level, there are patches or confluent zones of necrosis of the parenchymal acinar cells, together with foci of bleeding, necrosis of the vessel walls, and rupture of the pancreatic ducts. Additionally, significant inflammation and extensive intrapancreatic fat necrosis are the main characteristics [5].

This study was designed to examine the causes, various clinical manifestations, radiographic examination, and correlation between AP severity and prognosis as well as biochemical findings and consequences.

## Materials and methods

From January 2015 to December 2022, this retrospective investigation was carried out at the multidisciplinary industrial hospital in Jamshedpur, Jharkhand. A total of 1316 patients with pertinent symptoms were included in the study, and any of the two parameters, clinical, biochemical, and radiological assessment, confirmed the diagnosis of AP. Chronic pancreatitis patients were not included in this study. The study included radiological findings such as CT abdomen plain and contrast or ultrasonography (USG) abdomen confirming the presence of AP, biochemical parameters such as elevated serum amylase and lipase levels, and clinical manifestations such as acute abdominal pain and tenderness suggestive of pancreatitis. In this study, the severity of AP is determined based on the revised Atlanta Classification of Acute Pancreatitis (Table 1). Organ failure is defined in accordance with the Marshall scoring system as a score ≥ 2 for at least one of these three organ systems: respiratory, renal, and cardiovascular.

**Table 1:** Revised Atlanta Classification Source: Ref. [1].

Revised Atlanta Classification
A. Mild acute pancreatitis
(i) No organ failure
(ii) No local or systemic complications
B. Moderately severe acute pancreatitis
(i) Organ failure that resolves within 48 h (transient organ failure) and/or
(ii) Local or systemic complications without persistent organ failure
C. Severe acute pancreatitis: persistent organ failure (>48 h)
(i) Single organ failure
(ii) Multiple organ failure

All patients were treated with fluid resuscitation, support for the organ systems, pain relief, and nutritional assistance. For a possible pancreatic necrosis infection, antibiotics were taken. In cases with chronic organ failure (OF), probable infected necrosis, and/or pressure sensations, fluid collections were drained endoscopically or percutaneously. Those patients who did not improve after receiving medical care and drainage of collections required surgical necrosectomy.

Detailed history and investigations were carried out to identify the etiology of acute-on-chronic pancreatitis (ACP) including liver function test (LFT), fasting triglyceride, serum calcium, parathyroid hormone (PTH), and abdominal USG. All patients without contraindications for CT scans were subjected to contrast-enhanced CT scans of the abdomen, and specific features like collections and vascular complications were recorded. When the diagnosis remains elusive after the preliminary investigations, patients underwent advanced forms of investigation like magnetic resonance cholangiopancreaticography (MRCP). Genetic testing for hereditary pancreatitis was not carried out in our study.

Microsoft Excel/SPSS software was used to conduct the statistical analysis. During data analysis, the student t-test was used to compare continuous variables; the Chi-square test was used to compare dichotomous variables, and descriptive statistics were utilized as needed. The Fisher's exact test and Chi-square test were used to examine distributional differences. A p-value of 0.05 was deemed statistically significant. Before including patients in our study, informed consent was obtained from them.

## Results

In the present study, 1316 cases were enrolled. The most common age group was 30-44 years, i.e., 411 (31.23%) followed by 45-59 years, i.e., 357 (27.13%). The mean and median ages were 44.54 and 47 years, respectively (Table 2).

**Table 2:** Agewise distribution of patients with acute pancreatitis SD: Standard deviation.

Age in years	Frequency	Percentage
0–14	23	01.75%
15–29	167	12.69%
30–44	411	31.23%
45–59	357	27.13%
60–74	272	20.69%
75–89	83	06.30%
90+	03	00.23%
Total	1316	100.00%
Mean age ± SD	44.54 ± 28.98	

**Figure 1:**
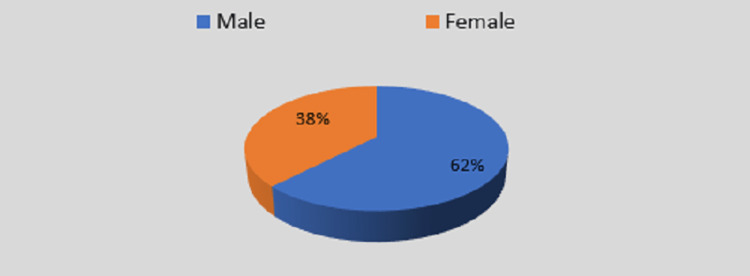
Gender distribution of selected patients with acute pancreatitis

The majority of cases were males (820 [62%]) in comparison to females (496 [38%]) as shown in Figure 1.

**Figure 2:**
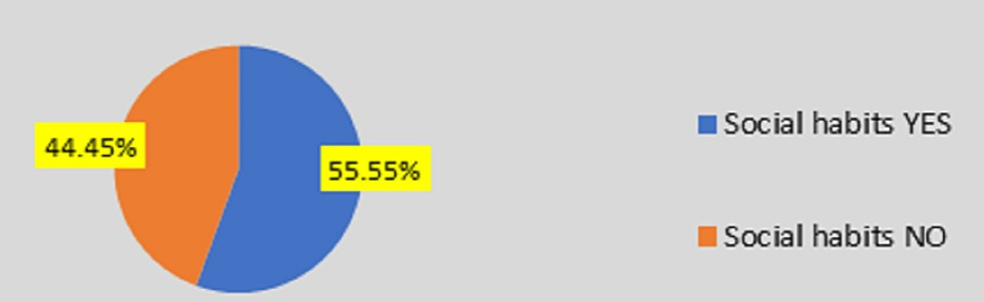
Distribution based on social habits in the selected acute pancreatitis patients

Based on social habits like alcoholism and/or smoking, most patients (731 [55.45%]) had a history of alcoholism and smoking (Figure 2).

Based on the presenting complaints, most patients were having abdominal pain (1262 [95.90%]) followed by vomiting (1163 [88.37%]), and 188 (14.28%) patients presented with yellowish discoloration of skin or eyes. Similarly, as per abdominal signs, the majority of patients were having epigastric tenderness (1237 [93%]) as shown in Table 3. Here, the most presenting complaints and abdominal signs overlapped between themselves.

**Table 3:** Presenting symptoms and abdominal findings of the selected acute pancreatitis patients The presenting complaints and abdominal signs were overlapping.

Variables	Frequency	Percentage
Presenting complaints		
Abdominal pain	1262	95.90%
Vomiting	1163	88.37%
Fever	331	25.16%
Abdominal fullness	276	20.10%
Yellowish discoloration of eyes/skin	188	14.28%
Abdominal signs		
Tenderness over the epigastric region	1237	93.00%
Abdominal distension	276	20.00%
Free fluid in the abdomen	89	06.76%
Lump in the abdomen	217	16.49%
Hepatomegaly	134	10.18%
Splenomegaly	121	09.19%

**Figure 3:**
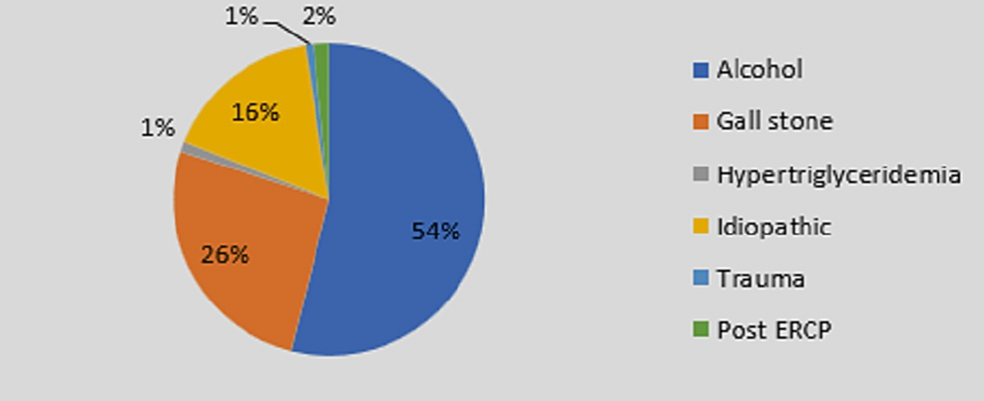
Distribution based on etiology ERCP: Endoscopic retrograde cholangiopancreatography.

The most common etiology of AP was alcoholism (710 [53.95%]) followed by gallstones (343 [26.06%]). Idiopathic pancreatitis was seen in 217 cases (16.48%) as shown in Figure 3.

Based on ultrasound findings, the majority of patients were having bulky pancreas (1052 [79.94%]) followed by gallbladder stones (343 [26.03%]) as shown in Table 4.

**Table 4:** Distribution based on USG findings in acute pancreatitis patients Test findings of USG were overlapping. USG: Ultrasonography; CBD: Common bile duct; GB: Gallbladder.

USG findings	Frequency	Percentage
Bulky pancreas	1052	79.94%
Peripancreatic collections	158	12.01%
Altered echotexture	263	19.99%
Fatty liver	168	12.76%
GB stone	343	26.03%
CBD stone	29	02.20%
Ascites ± pleural effusion	26	01.97%
Test not performed	89	06.76%

Based on the revised Atlanta classification of Acute Pancreatitis, most patients showed mild pancreatitis followed by moderate and severe pancreatitis, i.e., 937 (71.20%), 312 (23.71%), and 67 (05.09%), respectively. In 85 and 28 cases of mild and moderate pancreatitis, underlying features of chronic pancreatitis were present. In 19 cases of severe AP, hypocalcemia + shock + multi-organ dysfunction syndrome (MODS) features were seen (Table 5).

**Table 5:** Correlation between classifications of acute pancreatitis with complications *OF: Organ failure; MODS: Multi-organ dysfunction syndrome.

Classification of pancreatitis (Revised Atlanta Classification)	Complications of acute pancreatitis						Total
	No OF* and local or systemic complications	Ascites ± pleural effusion	Chronic pancreatitis	Hypocalcemia	Hypocalcemia + shock + MODS	Obstructive jaundice	
Mild	824	21	85	0	0	7	937
Moderate	279	4	28	0	0	1	312
Severe	38	1	0	9	19	0	67
Total	1141	26	113	9	19	8	1316

In mild, moderate, and severe AP, the mortality rates were 19 (02.03%), 44 (14.10%), and 21 (31.34%), respectively. Overall, 1232 (93.62%) AP cases recovered and were discharged in stable condition, but 84 (06.38%) cases expired (Table 6).

**Table 6:** Correlation between the severity of acute pancreatitis with outcomes

Crosstab count			
Classification of pancreatitis	Outcomes		Total
	Survived	Died	
Mild	918 (97.97%)	19 (02.03%)	937 (71.20%)
Moderate	268 (85.90%)	44 (14.10%)	312 (23.71%)
Severe	46 (68.66%)	21 (31.34%)	67 (05.09%)
Total	1232 (93.62%)	84 (06.38%)	1316 (100.00%)

A multivariate analysis of various biochemical factors with the classification of AP and the overall outcome was done. The highest mortality was seen with serum bilirubin > 1.2 mg/dl, serum amylase and lipase values exceeding 200 IU/l, and total leukocyte count (TLC) exceeding 15,000/mm^3^. Statistical differences were significant (p-value < 0.05) for all the mentioned parameters in Table 7.

**Table 7:** Results of multivariate analysis of factors affecting the classification of acute pancreatitis and outcomes TLC: Total leukocyte count; ALT: Alanine transaminases; S: Serum.

Variables	Values	Frequency	Classification of pancreatitis			Outcomes		p-values
			Mild	Moderate	Severe	Survived	Died	
S amylase	≤200 IU/L	237	171	44	16	218	19	<0.05
	>200 IU/L	1079	766	268	51	1014	65	<0.05
S lipase	≤200 IU/L	273	195	61	17	250	23	<0.05
	>200 IU/L	1043	742	251	50	982	61	<0.05
TLC	≤15,000/mm^3^	435	231	80	13	417	18	<0.05
	>15,000/mm^3^	881	706	232	54	815	66	<0.05
ALT	≤45 IU/L	230	158	58	14	213	17	<0.05
	>45 IU/L	1086	779	254	53	1019	67	<0.05
S. bilirubin total	≤1.2 mg/dl	169	109	51	09	158	11	<0.05
	>1.2 mg/dl	1147	828	261	58	1074	73	<0.05

## Discussion

This was a retrospective study consisting of 1316 cases of AP. AP is a relatively common disease with an incidence of 5-80 per 100,000 members of the population worldwide. Although its prevalence varies in different countries and even in various areas of a given country, there has been a significant increase in the number of new cases in recent years [6]. This study shows that the most common age group was 30-44 years (411 [31.23%]), followed by 45-59 years (357 [27.13%]), and the mean and median ages were 44.54 and 47 years, respectively.

Reddy et al. [6] showed that out of 60 AP cases, the majority of the patients (28 [46.6%]) were in the age group of 15-30 years, 26 (43.3%) were in the age group of 30-45 years, 3 (5%) were in the age group of 45-60 years, and 3 (5%) were in the age group of 60-75 years. However, Nandu et al. [7] found that the highest incidence was noted in patients in the age group of 20-40 years, accounting for 52.11% of patients, and the mean age of presentation was 38.94 years. Also, Jha et al. [8] found that the mean age of the study group (n = 104) was 40.9 ± 1.3 years, and 104 patients were grouped in ages of <25, 25-35, 36-45, 46-55, and >55 years. The majority of patients were in the age group of 36-45 years with AP. Similarly, Ahlawat et al. [9] found that the mean age of the study sample (n = 50) was 47.30 ± 15.16 (SD) years, ranging from 19 to 75 years. In a study by Das et al. [10], 63% of patients were in the age group of 20-39 years, 28% in 40-59 years, 4% in <20 years, and 5% in 60-80 years. In the present study, the majority of cases (62%) were males. Similar findings were found in the studies by Reddy et al. [6], Nandu et al. [7], and Das et al. [10], i.e., 95%, 92.25%, and 96%, respectively. However, Jha et al. [8] and Ahlawat et al. [9] found that the majority of cases were females, i.e., 65% and 70%, respectively.

Based on social habits like alcoholism and/or smoking, most patients (731 [55.45%]) were having a history of alcoholism and smoking in this study. Similar to our study, Reddy et al. [6] showed that in a total of 60 patients, 57 (95%) patients had social habits (i.e., alcohol and smoking), and three (5%) patients were not having any social habits. Our study found abdominal pain was the most common presenting complaint (95.90%). Reddy et al. [6], Nandu et al. [7], and Das et al. [10] found abdominal pain as a major presenting complaint (100%), followed by Ahlawat et al. (98%) [9]. Reddy et al. [6], Nandu et al. [7], and Kalyani et al. [11] showed that alcohol is the most common etiology for AP, but Jha et al. [8] and Ahlawat et al. [9] found gallstone as the major etiology for the same.

In our study, ultrasound findings revealed that patients were having bulky pancreas (1052 [79.94%]) followed by gallbladder stones (343 [26.03%]). Similarly, Reddy et al. [6] showed that 67% of patients had a bulky pancreas and 45% had altered echo texture. Table 8 shows a comparison of the severity of AP and their relative percentage, and the results are comparable to the present study.

**Table 8:** Distribution of the severity of acute pancreatitis patients with different studies AP: Acute pancreatitis.

Studies	Mild AP	Moderate AP	Severe AP
Present study	71.20%	23.71%	05.09%
Reddy et al. [6]	70%	30%	00%
Nandu et al. [7]	77.40%	00%	22.60%
Jha et al. [8]	77.80%	00%	22.20%
Ahlawat et al. [9]	82%	00%	18%
Das et al. [10]	83%	14%	03%
Patel et al. [12]	31.70%	25.00%	43.30%
Pongprasobchai et al. [13]	72%	16%	12%

The multivariate analysis of various biochemical factors with the classification of AP and the overall outcome is also supported by Reddy et al. [6], Matull et al. [14], and Esmaili et al. [15]. Our study showed a mortality rate of 06.38%. Nandu et al. [7], Ahlawat et al. [9], Ahmed et al. [4], Reid et al. [16], and Anderson et al. [17] showed that the mortality in AP was 04.34%, 4%, 6%, 2%, and 9%, respectively.

In the present study, when the multivariate analysis of various biochemical factors with the classification of AP and the overall outcome was done, the highest mortality was seen with serum bilirubin > 1.2 mg/dl, serum amylase and lipase values exceeding 200 IU/l, and TLC exceeding 15,000/mm^3^. Similar to this, Reddy et al. [6] demonstrated that the majority of patients had elevated serum amylase levels, with 32 (53.3%) patients having levels between 200 and 450, 22 (36.7%) patients having levels between 450 and 900, and six (10%) patients having levels between 140 and 200 IU/L. The majority of the patients, 29 (48.4%), ranged from 200 to 450, 18 (30%) ranged from 450 to 900, and 13 (21.6%) ranged from 160 to 200 IU/L due to the overactivation of the amylase enzyme inside the acinar cells as a result of this. This further suggests overactivation of the lipase enzyme inside the acinar cells and also results in pancreatic autodigestion, which is related to the severity of the condition and ultimately reveals negative results.

One of the limitations of our study was that we did not do a genetic analysis to identify the cause of pancreatitis. This research also had a relatively small patient population and was done at a single hospital. This study will encourage researchers to carry out a multicenter study with a sizable sample of patients in order to learn more about the epidemiology, clinical characteristics, and outcomes of individuals with AP.

## Conclusions

Adults and patients in their third decade were found to have the highest prevalence of AP. In the oldest and youngest age groups, it was comparatively less prevalent. Males were shown to have a higher incidence of AP than females. This could be explained by the increased prevalence of alcohol addiction among men in this region of the world. We come to the conclusion that poor patient outcomes were associated with serum lipase values > 200 IU/L, TLC > 15000/mm^3^, and the emergence of complications such as ascites, pancreatic necrosis, and OF. For early intensive therapy, prompt intervention, and enhancing the quality of life, prognosis, and survival, early assessment of the clinical severity and identification of people at risk are crucial.
